# Binational patterns in use of Food is Medicine among Mexican immigrants

**DOI:** 10.3389/fnut.2026.1810991

**Published:** 2026-04-24

**Authors:** Marcela D. Radtke, Adriana Maldonado, Caroline Owens, Lan Xiao, Daniel E. Martínez, David Garcia, Lisa G. Rosas, Rebecca Crocker

**Affiliations:** 1 Department of Epidemiology and Population Health, Stanford University School of Medicine, Stanford, CA, United States; 2 Mel and Enid Zuckerman College of Public Health, University of Arizona, Tucson, AZ, United States; 3 Department of Anthropology, University of North Carolina Wilmington, Wilmington, NC, United States; 4 Food is Medicine Institute, Tufts University, Medford, MA, United States; 5 Office of Community Engagement, Stanford University School of Medicine, Stanford, CA, United States

**Keywords:** binational migration, dietary intake, Food is Medicine, health-related foods, Mexican immigrant, nutrition transition

## Abstract

**Introduction:**

Migration may affect how immigrants use foods and beverages to prevent or treat acute and chronic illness; however, little is known about the impact of binational migratory patterns on medicinal food and beverage intake. Characterizing food and beverage intake pre- and post-migration is critical for informing culturally-centered nutrition programs, such as Food is Medicine (FIM).

**Objective:**

To characterize changes in reported medicinal food and beverage intake and ethnomedicinal classifications used to prevent or treat illness pre- and post-migration from Mexico to the United States (US).

**Methods:**

A retrospective, cross-sectional study was conducted using a purposive community-based survey of Mexican immigrants living in the US (*n* = 300). Reported medicinal food and beverages consumed to prevent or treat illness were categorized by major organ system. McNemar test or McNemar exact test for low counts were conducted to examine changes in foods and beverages pre- and post-migration.

**Results:**

Most participants reported consuming soups and stews for medicinal purposes both when living in Mexico (75.9%) and after migrating to the US (72.7%). No statistically significant differences were observed between foods and beverages consumed for medicinal purposes pre- and post-migration, with the exception of herbs, which were consumed more prior to migration. Foods and beverages were most used to address overall health and wellness, respiratory conditions (i.e., colds, flus, coughs), and digestive conditions (i.e., stomach pain, diarrhea, indigestion). Following migration to the US, participants began to emphasize the prevention of nutrition-sensitive chronic diseases, such as diabetes, as part of their broader health concerns.

**Conclusion:**

Findings from this study suggest that while medicinal foods did not significantly differ following migration, except for herbs, some of the health-related reasons shifted away from acute illnesses, such as respiratory and digestive conditions, toward preventing nutrition-sensitive chronic diseases. The integration of cultural knowledge and ethnomedicinal beliefs related to food may be used to inform and strengthen nutrition interventions.

## Introduction

Food is Medicine (FIM), or the integration of nutrition programs into healthcare, is increasingly implemented to address the rising burden of nutrition-sensitive chronic conditions and social drivers of health (1). As part of efforts to develop and implement culturally-centered FIM programs, it is critical to understand how diverse groups, such as immigrant communities, use foods to prevent, manage, and treat disease. Throughout history, cultures worldwide have leveraged locally available natural resources for medicinal and therapeutic purposes (2). These ethnomedicinal beliefs, practices, and cultural ideas about health and healing often include foods and beverages with bioactive properties (3). Gaining a deeper understanding of ethnomedicinal beliefs can inform culturally tailoring of FIM programs, including what foods are offered and the content of accompanying nutrition education.

Ethnomedicinal practices are common among the largest immigrant group in the US, people from Mexico, who comprised 23% of the immigrant population in 2023 (4). In Mexico, the consumption of ancestral or traditional foods is widely practiced as a strategy for treating acute illness and preventing diseases (5, 6). The components of ancestral or traditional Mexican diets vary across geographical regions of Mexico, and include a wide variety of foods including grains, legumes, tubers, maize (corn) products, meat, fruits, and vegetables (6, 7). Many of these foods are naturally rich in bioactive compounds, such as fiber, polyphenols, and carotenoids, which are known to increase anti-inflammatory response cascades and antioxidant capacity to support immune function to reduce the severity of illness and chronic disease risk (8, 9). Evidence from the 2018–2019 National Health and Nutrition Survey (ENSANUT) in Mexico provides support for the health-promoting characteristics of the traditional Mexican diet, where adults with high adherence to a traditional Mexican diet had significantly lower insulin and total, low-density lipoprotein (LDL), and non-high-density lipoprotein (HDL) cholesterol concentrations (10).

Migration can profoundly influence these ethnomedicinal practices, including food preferences, choices, and consumption (11, 12). Following migration from Mexico to the US, local sociodemographic characteristics and established social networks have the capacity to re-shape food-related behaviors and diet-health associations. Migration-related challenges, including language barriers, financial constraints, and commercial availability, may limit access to foods traditionally consumed for medicinal purposes (13). In border regions, such as Southern Arizona, dietary practices among Mexican immigrants are embedded within a broader set of health-related behaviors that are shaped by place-based factors operating on both sides of the US-Mexico border (14, 15). Place-based factors along the US-Mexico border can support or hinder health-promoting dietary practices (16). Supportive factors may include a high density of Mexican grocery stores in US border states or engagement with community health workers (known in Spanish as *promotoras*), who are embedded within the target communities and share cultural knowledge, lived experiences, sociocultural norms, and native Spanish language skills (17). Conversely, some place-based factors may result in structural barriers to medicinal dietary practices, including exclusion from federal nutrition assistance programs, such as the Supplemental Nutrition Assistance Program (SNAP), limited access to healthcare-based nutrition counseling, and broader systemic factors, such as food insecurity (18). In addition to place-based factors, longer residence in the US may shape changes in food-related beliefs and practices, reflecting alignment with dominant cultural norms (19). Despite these factors, Mexican immigrants often maintain culturally rooted dietary behaviors acquired in their country of origin, while simultaneously adapting to new food environments in the US.

Building on existing evidence that dietary practices are shaped by both cultural heritage and migration, this study examines how Mexican-born immigrants living in Southern Arizona utilize foods and beverages for medicinal purposes before and after migration. The primary objectives were to identify and characterize the illnesses that were considered preventable and amendable through dietary practices among Mexican-born immigrants, and to explore potential changes in dietary intake of medicinal foods and beverages pre- and post-migration. Findings from this ethnomedicinal analyses can be used to inform the design and implementation of culturally-centered nutrition interventions, including FIM programs.

## Materials and methods

### Study design

This secondary analysis uses data from a retrospective cross-sectional study that was conducted to document health and health care experiences in Mexican-origin adults in Southern Arizona before and after migration (20). The details of this study among first-generation Mexican immigrants have been previously published (20, 21). In brief, a telephone-based survey was conducted from April 2022 to February 2023 in the participants’ preferred language (Spanish; *n* = 297 or English; *n* = 3). Participants were eligible for inclusion if they met the following criteria: 1. Over the age of 18 years; 2. Born in Mexico; 3. Immigrated to the United States at age 15 or older; and 4. Currently lived in Southern Arizona. The study was approved by the Institutional Review Board at the University of Arizona (Protocol ID: 00000368) and verbal consent was acquired in the participant’s preferred language prior to participation in study activities.

### Variables

This analysis used survey questions related to foods and beverages consumed pre- and post-migration and the specific illnesses for which they were consumed. The questions regarding traditional foods consumed for medicinal purposes were separated into two parts. First, participants were primed to retrospectively reflect on foods and beverages consumed prior to migrating to the US with the following question: “Before migrating to Arizona, did you and your family ever consume particular foods and beverages to prevent or treat any illnesses in Mexico, or not? Participants with affirmative responses where then asked subsequent questions to identify the foods and beverages with their associated illnesses: “Considering up to five, what foods and beverages did you consume to prevent or treat illnesses when living in Mexico?”; and “For which illnesses did you consume them?” Participants were then asked to respond to the following questions in the present, post-migration period: “Considering up to five, what foods and beverages do you consume to prevent or treat illnesses in Arizona?”; and “For which illnesses do you consume them?” The use of open-ended survey questions was designed so that participants were able to list up to five foods and their associated illnesses/sicknesses and to reduce potential for agreement biases associated with multiple-choice format (22). Participant responses were translated into English, except when foods or meals did not have a known translation (i.e., atole: a corn flour masa-based drink made from milk or water and served hot, with significant regional variations). Data were categorized by pre- or post-migration.

### Statistical analysis

Descriptive statistics were expressed as mean and standard deviations (SD) or by the sample size (n) and percentages (%). Open-ended responses were reviewed and coded independently by two bilingual, bicultural researchers, with discrepancies resolved through discussion until consensus was reached between the coders. Foods, mixed dishes, and beverages were categorized into the following: soups and stews, cold drinks, hot drinks, meat, seafood, grains, vegetables, fruit, legumes, herbs, and other animal-based foods, such as eggs, dairy products, and honey (Supplementary Table 1). Pairwise comparisons were performed using McNemar test or McNemar exact test for low counts to examine changes in foods and beverages consumed pre- and post-migration. Analyses were stratified by biological sex and age at migration. Generalized linear mixed models with repeated measures were constructed to assess the differences between pre- and post-migration for female and male, and between pre- and post-migration for age greater than and equal to 30 and less than 30, as these sociodemographic factors have previously demonstrated effects on health-related beliefs and behaviors, including those related to diet (21).

**Figure 1:**
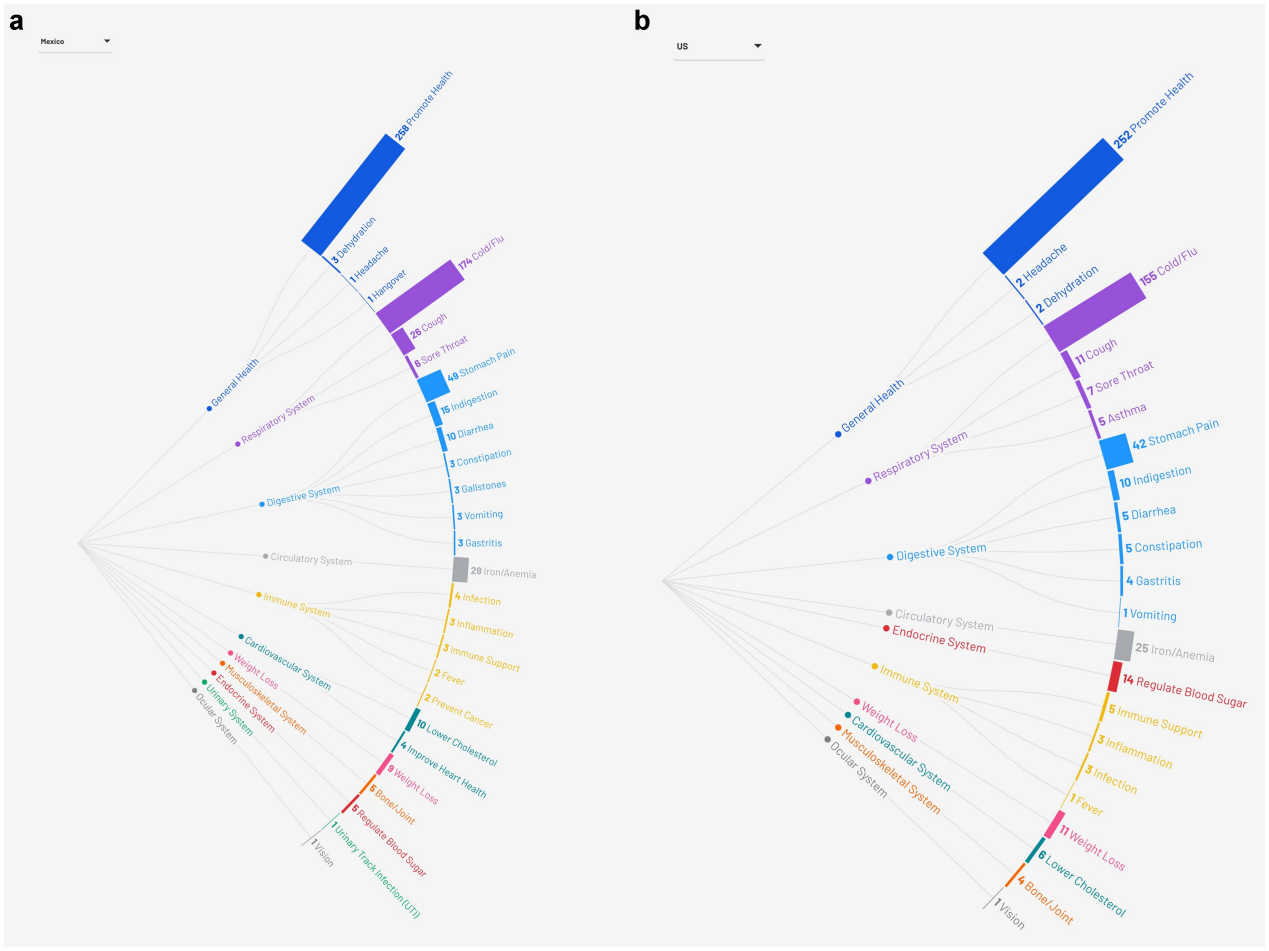
**(a)** Categorization of acute and chronic illness reported pre-migration to be preventable or amendable through dietary practices into major organ systems. **(b)** Categorization of acute and chronic illness reported post-migration to be preventable or amendable through dietary practices into major organ systems.

**Figure 2:**
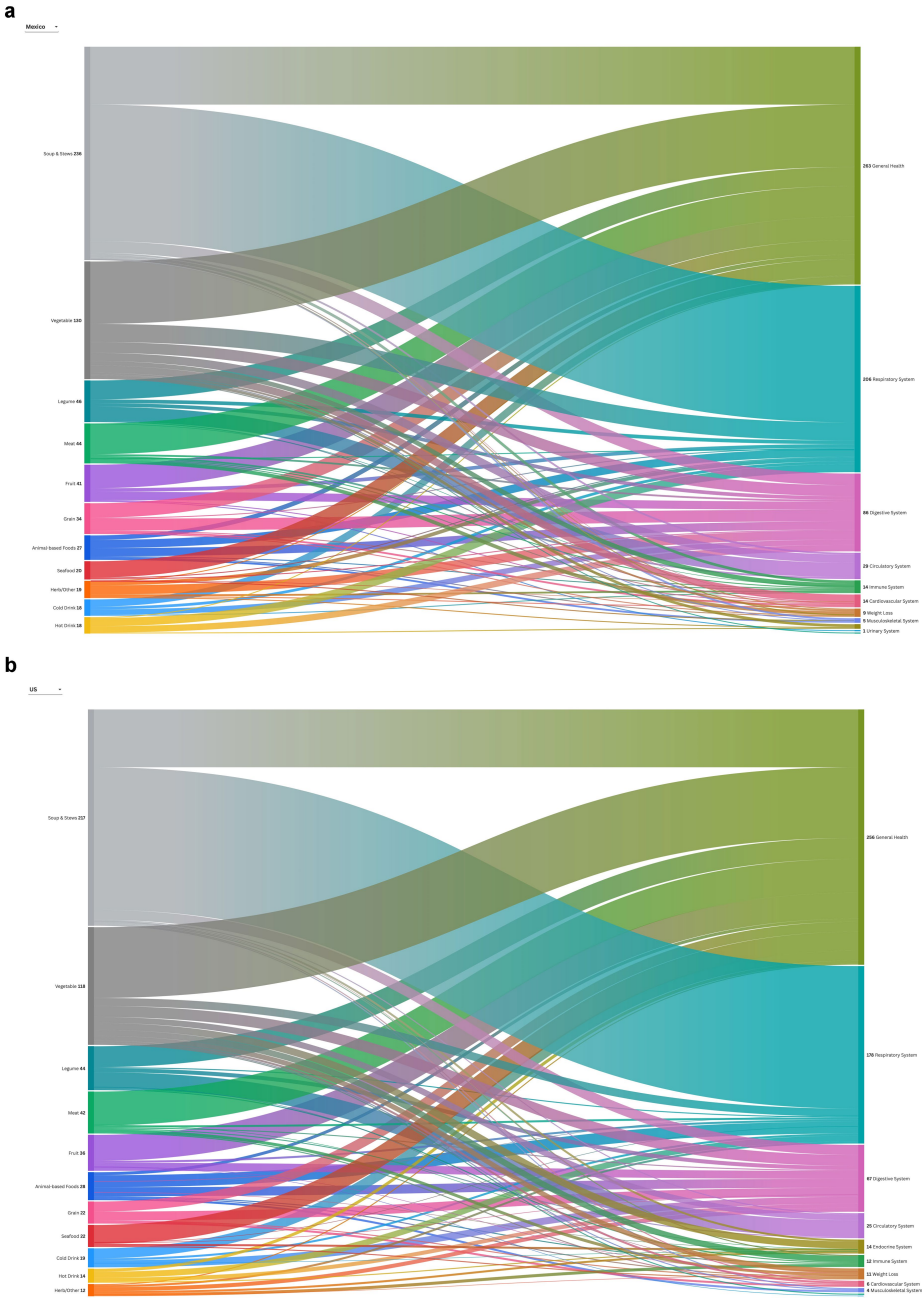
**(a)** Sankey diagram of the foods reported pre-migration to prevent and treat illness. **(b)** Sankey diagram of the foods reported post-migration to prevent and treat illness.

The associated illnesses for which participants reported consuming food and beverages were similarly categorized independently by two reviewers based on the major organ systems of the human body and discrepancies were reconciled through discussion (23). For example, illnesses such as a cough, cold, and flu were aggregated into conditions that affect the respiratory system and depicted graphically in a radial tree (Figures 1a,b). When a food was described as being used to treat more than one illness, it was coded to reflect each reported medicinal use and therefore the frequencies are not mutually exclusive. A Sankey diagram was constructed to visualize patterns in the use of medicinal foods and beverages and the associated illness categories pre- and post-migration (Figures 2a,b). No adjustments for multiple hypothesis testing were applied, as such the analyses were exploratory in nature and intended to identify potential patterns. Statistical analysis was performed in SAS version 9.4 (SAS Institute Inc., Cary, North Carolina) and statistical significance was defined at *p* < 0.05 (two-sided). Graphics were created in Flourish.^1^

## Results

Participants were Mexican-born adults residing in Southern Arizona (*n* = 300), primarily female (77.3%) with a mean age of 49.5 ± 13.0 years (Table 1). Most participants were married (69.3%), with an average monthly income of $2,376 ± $1,751. Participants migrated to the US at a mean age of 29.8 ± 12.1 years and lived in the US for approximately 17.9 ± 12.0 years, with an immigration status of documented non-citizen (48.6%), US citizen (32.9%), and undocumented non-citizen (18.5%). Out of the total sample, 72 participants reported no medicinal food or beverage use prior to migration and 80 participants reported no use post-migration.

**Table 1:** Baseline characteristics of Mexican immigrant participants in the Salud sin Fronteras cohort.

Participant characteristics	Mean (SD) or %
Sociodemographic Characteristics (*n* = 300)	
Age, years (mean, SD)	49.5 (13.0)
18–34 yrs	11.3
35–49 yrs	41.7
50–64 yrs	32.0
65 + yrs	15.0
Biological sex	
Female	77.3
Male	22.7
Birth place	
Sonora	70.3
Sinaloa	7.3
Chihuahua	3.0
Distrito federal	3.0
Jalisco	2.7
Other	13.7
Birth place size	
Large City	48.0
Small city/town	47.3
Colonia/informal settlement	0.7
Rancho	2.7
Ejido	1.0
Do not know	0.3
Marital status	
Married	69.3
Single	11.7
Divorced/Separated	9.4
Widowed	7.3
Civil Union	2.3
Current household size	3.5 (1.6)
1–2 people	32.0
3–5 people	58.7
6–10 people	9.3
Socioeconomic characteristics	
Educational attainment	
Less than High School	29.7
High School or GED	27.0
Some College, no degree	15.0
College degree or higher	28.3
Employment status	
Yes, full-time	30.3
Yes, part-time	19.7
No, but on disability, unemployment, or retirement	8.6
No, no source of income	41.3
Monthly household income ($) (*n* = 275)	$2,376.8 ($1,751.4)
$0–999	13.5
$1,000–1,999	25.5
$2,000–2,999	33.8
$3,000–3,999	12.0
$4,000+	15.3
Currently have health insurance? (*n* = 298)	
Yes	56.4
Immigration-related factors	
English Fluency	
Fluent	6.4
Very well	12.0
Well	23.8
Not very well	18.4
Does not speak English	39.5
Migration Age (yrs)	29.8 (12.1)
0–17 yrs	11.0
18–24 yrs	29.3
25–34 yrs	30.0
35–49 yrs	21.0
50 + yrs	8.7
Years in the US (yrs)	17.9 (12.0)
0–10 yrs	32.3
11–20 yrs	32.0
21–30 yrs	22.3
31–40 yrs	8.0
41 + yrs	5.3
Immigration status (*n* = 286)	
Undocumented noncitizen	18.5
Documented noncitizen	48.6
US citizen	32.9

Prior to migration to the US, participants reported 472 foods and beverages that were consumed for managing 633 various illnesses when reflecting on their experience living in Mexico. The most commonly consumed foods included soups and stews (75.9%), vegetables (38.2%), legumes (17.1%), fruit (14.9%), and meat (14.0%; Table 2). The primary purpose for consumption of medicinal foods was to promote overall health and wellness (41.6%; Figure 2a). Soups and stews, specifically chicken soup (caldo de pollo), vegetables, and hot drinks (i.e., tea and atole), were the primary medicinal foods consumed to treat colds and flus (32.5%). Illness associated with the digestive system (13.6%), such as stomach pain, indigestion, diarrhea, gastritis, gallstones, and constipation, were managed with soups and stews, grains, and vegetables. Other health concerns included iron deficiency and anemia (4.6%), for which legumes, such as beans and lentils, and vegetables, including squash, spinach, and beets, were primarily consumed.

**Table 2:** Pairwise comparisons of the foods used among Mexican immigrants to prevent and treat illness pre- and post-migration.

Foods		Geographic location		
English	Spanish	Mexico (*n* = 228)	Arizona (*n* = 220)	*p*-value^a^
		n (%)	n (%)	
***Soups and Stews***	***Sopas y Caldos***	173 (75.9)	160 (72.7)	0.45
Chicken	Pollo	133 (58.3)	114 (51.8)	0.17
Red Meat	Carne	15 (6.6)	17 (7.7)	0.64
Cheese	Queso	2 (0.9)	4 (1.8)	0.39
Vegetable	Verdura	22 (9.6)	18 (8.2)	0.59
Pasta	Fideo	2 (0.9)	2 (0.9)	0.97
Marisco	Marisco	3 (1.3)	2 (0.9)	0.68
Other Soups	Otras sopas	41 (18)	45 (20.5)	0.51
***Cold Drinks***	***Bebidas Frias***	17 (7.5)	18 (8.2)	0.77
Rice milk	Leche de Arroz/Horchata	5 (2.2)	4 (1.8)	0.78
Lemon	Limon	2 (0.9)	1 (0.5)	0.58
Hibiscus	Jamaica	1 (0.4)	0 (0.0)	
Tamarind	Tamarindo	0 (0.0)	0 (0.0)	
Papaya	Papaya	1 (0.4)	1 (0.5)	0.98
Other cold drinks	Ortas bebidas frias	8 (3.5)	14 (6.4)	0.16
***Hot Drinks***	***Bebidas Calientes***	11 (4.8)	11 (5)	0.93
Chamomile	Manzanilla	1 (0.4)	0 (0.0)	
Mint	Menta	1 (0.4)	0 (0.0)	
Spearmint	Hierbabuena	0 (0.0)	2 (0.9)	0.15
Corn-based drink	Atole/champurrado/pinole	6 (2.6)	3 (1.4)	0.34
Other hot drinks	Ortas bebidas calientes	5 (2.2)	7 (3.2)	0.52
***Meat***	***Carne***	32 (14)	31 (14.1)	0.99
Beef	Carne de res	11 (4.8)	7 (3.2)	0.38
Chicken	Pollo	15 (6.6)	20 (9.1)	0.32
Organ meat	**Vísceras**	4 (1.8)	2 (0.9)	0.44
Other meats	Otras carnes	6 (2.6)	7 (3.2)	0.73
***Seafood***	***Marisco***	17 (7.5)	18 (8.2)	0.77
***Cereal***	***Cereales***	23 (10.1)	21 (9.5)	0.85
Corn	Maiz	2 (0.9)	2 (0.9)	0.97
Oats	Avena	9 (3.9)	6 (2.7)	0.47
Rice	Arroz	11 (4.8)	9 (4.1)	0.71
Wheat	Trigo	1 (0.4)	1 (0.5)	0.98
Other cereals	Otros cereales	1 (0.4)	3 (1.4)	0.30
***Vegetables***	***Verduras***	87 (38.2)	80 (36.4)	0.69
Greens	Quelite	14 (6.1)	7 (3.2)	0.14
Potatoes	Papas	9 (3.9)	6 (2.7)	0.47
Onion	Cebolla	7 (3.1)	6 (2.7)	0.83
Garlic	Ajo	4 (1.8)	6 (2.7)	0.49
Beets	Betabel	4 (1.8)	2 (0.9)	0.44
Squash	Calabaza/Chayote	1 (0.4)	3 (1.4)	0.30
Nopales (cactus)	Nopales	13 (5.7)	9 (4.1)	0.43
Salad	Ensalada	8 (3.5)	12 (5.5)	0.32
Other vegetables	Otras verduras	56 (24.6)	58 (26.4)	0.66
***Fruit***	***Fruta***	34 (14.9)	33 (15)	0.98
Lemon	Limon	7 (3.1)	3 (1.4)	0.22
Papaya	Papaya	3 (1.3)	2 (0.9)	0.68
Orange	Naranja	6 (2.6)	2 (0.9)	0.17
Other fruits	Otras frutas	24 (10.5)	29 (13.2)	0.38
***Legumes***	***Legumbre***	39 (17.1)	34 (15.5)	0.64
Beans	Frijoles	28 (12.3)	21 (9.5)	0.35
Lentils	Lentejas	13 (5.7)	18 (8.2)	0.30
Chickpea	Garbanzo	2 (0.9)	5 (2.3)	0.23
Other legumes	Otros legumbres	0 (0.0)	0 (0.0)	
***Herbs***	***Hierbas***	15 (6.6)	7 (3.2)	**0.04**
***Animal-based Foods***	***Comidas de Animales***	24 (10.5)	25 (11.4)	0.78
Honey	Miel	9 (3.9)	9 (4.1)	0.94
Eggs	Huevos	6 (2.6)	4 (1.8)	0.56
Dairy Products (milk, cheese, yogurt)	Productos Lactivos (leche, queso, yogur)	5 (2.2)	4 (1.8)	0.78
Gelatin/Jello	Gelatina	6 (2.6)	9 (4.1)	0.39
Other animal-based foods	Otras Comidas de Animales	0 (0.0)	0 (0.0)	

a *p*-values were obtained from McNemar test or McNemar exact test for low counts for pairwise comparisons. Statistical significance (*p* < 0.05) is denoted by bold values.

Following migration to the US, participants reported consuming 438 foods for managing 574 illnesses. Similar to pre-migratory foods, the foods consumed in the US included soups and stews (72.7%), vegetables (36.4%), legumes (15.5%), fruit (15.0%), and meat (14.1%; Table 2). In parallel with the illnesses noted pre-migration, most of the foods consumed post-migration were noted to promote overall health (44.6%), reduce the severity and duration of colds and flus (31.0%), prevent stomach pain and gastric distress (11.7%), and address iron deficiency or anemia (4.4%; Figure 2b). Post-migration, participants reported consuming foods to address several nutrition-related chronic diseases, including concerns around preventing diabetes and obesity through the consumption of meat, seafood, and vegetables. The usage of medicinal foods to address nutrition-sensitive chronic diseases, including regulating blood sugar to prevent or treat type 2 diabetes, was higher post-migration (2.4%) compared to pre-migration (0.8%), although overall prevalence remained low (Figures 1a,b).

There were no statistically significant differences observed between the specific medicinal foods and beverages consumed for preventing or treating illnesses from pre- and post-migration, with the exception of herbs (Table 2). Herbs, including leaves and floral varietals such as oregano, mustard seed, and castor oil, were consumed significantly more for medicinal purposes pre-migration (6.6%) compared to post-migration (3.2%). Similarly, there were no differences in medicinal food practices by biological sex pre- and post-migration (Supplementary Table 2) nor the amount of time spent in Mexico prior to migration to the US (migration prior to 30 years of age vs. over 30 years of age), excluding quelites, which were consumed significantly more among participants who migrated earlier in life when in Mexico compared to the US (Supplementary Table 3). Quelites encompass an array of wild leafy green vegetables, including amaranth leaves, which were primarily consumed to prevent anemia, improve digestion, and promote overall health.

## Discussion

In this retrospective cross-sectional study of ethnomedicinal food practices among Mexican immigrants, no statistically significant differences were observed in the consumption of foods and beverages to treat illness from pre- to post-migration to the US, with the exception of herbs. The primary foods consumed among participants when living in both Mexico and the US were soups and stews, vegetables, legumes, meats, and fruit, aligning with the core components of a traditional Mexican dietary pattern (24). Medicinal dietary practices did not differ between male and female participants or by period of time living in Mexico prior to migrating to the US. The persistence of medicinal foods and beverages may reflect the larger demographic composition of Southern Arizona (over 30% Hispanic origin), which supports the accessibility and availability of traditional Mexican foods post-migration (25, 26). Given the proximity to the US-Mexico border, cross-border mobility may also facilitate the use of medicinal foods and beverages after migration, which aligns with a prior examination of traditional Mexican medicinal behaviors more generally, including over the counter medication use and herbal remedies, likewise promoted sustained cultural practices post-migration (27, 28).

The medicinal foods and beverages reported pre- and post-migration were consumed to address a multitude of illnesses, primarily emphasizing those that support overall health and wellness, the respiratory system, such as preventing or treating colds and flus, and the digestive system to address stomach pain, diarrhea, and indigestion. Descriptive analyses of the most frequently reported food–illness pairings pre- and post-migration indicated that soups and stews were commonly used to address respiratory illness, while vegetables were consumed to support general health. Although minor shifts in the specific foods reported for medicinal use were observed, participant responses suggest a continuity of culturally rooted practices, with adaptations reflecting the availability of familiar foods in new contexts. Following migration from Mexico to the US, a greater emphasis was placed on the use of foods, such as vegetables, meat, and seafood to address nutrition-sensitive chronic conditions, including diabetes (29, 30). This shift toward acknowledging the importance of diet in preventing and managing nutrition-sensitive chronic conditions is consistent with existing literature, that describes heightened susceptibility after migration, as greater visibility of diabetes risk within immigrant communities in the US may influence preventive dietary behaviors (31, 32).

The medicinal foods reported in this study aligned with the high dietary quality of a traditional Mexican diet, focused on nutrient-dense soups and stews, vegetables and fruits with diverse micronutrient and phytonutrient compositions, and lean meat and seafood-based protein sources (33, 34). This study specifically examined foods linked to preventing and treating illness, rather than assessing diet quality, as dietary intake among Mexican immigrants in the US has previously been explored (35, 36). The foods and beverages consumed for medicinal purposes remained relatively constant pre- and post-migration despite age at the time of migration and biological sex being previously associated with differences in dietary intake and diet patterns among Mexican immigrants (31). As the present analyses focused on immigrants who lived in Mexico for a minimum of 15 years prior to migrating to the US, the observed maintenance of foods consumed for medicinal purposes pre- and post-migration provides evidence in support of the enduring preservation of traditional cultural knowledge and practices (37, 38). Given that the study sample comprised predominantly of adults (≥18 years; 89%) who lived in the US for over 10 years (67.7%), the persistence of traditional foods and beverages consumed for medicinal purposes may reflect continuity in pre-migration health behaviors that support the attenuation of risk factors associated with nutrition-sensitive chronic diseases (39).

### Implications for practice

Informed by an ethnomedicinal approach to assess medicinal foods and beverages consumed pre- and post-migration, these findings support the expansion of context-specific culturally tailored nutrition interventions within US-Mexico border regions, such as Southern Arizona, as an effective approach for addressing nutrition-sensitive chronic conditions among Mexican immigrant populations (20, 40). Prioritizing the incorporation of medicinal foods traditionally used for the prevention and treatment of illnesses may enhance the cultural relevance, acceptability, and potential effectiveness of nutrition education and intervention efforts among Mexican-origin populations in border states. An example for such incorporation is within FIM interventions, which adopt a healthcare-integrated approach to prevent, manage, and treat nutrition-sensitive chronic diseases in healthcare and community settings (41). To date, few FIM programs include in-depth cultural tailoring beyond language translations (42). Including culturally concordant foods, recipes, and nutrition education, along with a place-based approach, in FIM programming may not only strengthen healthcare and community-based connections to nutrition services but also provide resources to maintain and preserve healthful cultural knowledge and practices within immigrant communities (43). Emerging evidence also suggests that, while sustained practices of Mexican traditional diet declines among subsequent generations of Mexican Americans, these populations may experience significant health benefits from traditional Mexican foods due to gene–environment interactions and epigenetic processes linked to ancestral dietary exposures (44). Therefore, to inform precision health approaches within FIM programming, there is an urgent need to engage Mexican communities, specifically foreign-born Mexican immigrants in similar border state environments, to determine which traditional foods and recipes should be included to increase resilience of cultural knowledge and promote long-term dietary behavior change (45).

Potential limitations in the wide-spread adoption of FIM interventions among immigrant populations are the extensive barriers in accessing healthcare services in the US, including administrative challenges, cost, and language proficiency (46, 47). As with the 43.6% of uninsured participants in this study, individuals without health insurance face structural and societal barriers to accessing clinical care. Community-engaged and community-centered FIM interventions, such as those delivered through community-based organizations, food pantries, or food banks, may provide an alternative approach to nutrition support outside of traditional healthcare settings (48, 49).

### Limitations

It is important to recognize the potential limitations in the present analyses. Due to the challenges in conducting longitudinal research in immigrant populations, a retrospective cross-sectional study design was employed to assess dietary behaviors pre- and post-migration (50, 51). This study design limits the ability to determine any causal relationships and introduces potential recall biases (52). As the study population reported living in the US for almost two decades (17.9 years), memory decay and the potential reconstruction of past behaviors based on current dietary practices may have introduced substantial biases, which should be considered when interpreting the findings. Open-ended survey responses were used to capture the diversity of foods and beverages consumed for medicinal purposes, providing robust data that directly linked foods with specific illnesses. Responding to open-ended questions generally requires greater cognitive effort than when responding to closed-ended questions, as participants are required to generate answers in their own words (53). As brief or incomplete responses were expected, the qualitative coders interpreted the referenced foods or illnesses to the extent possible when responses lacked sufficient detail or clarity. Of note, the qualitative coders were bilingual and bicultural, which enabled familiarity with foods and illnesses commonly referenced using colloquial or region-specific terminology (54). Given that the population was specific to Mexican immigrants living in Southern Arizona, the foods and associated illnesses may not be generalizable to other Hispanic migrant populations or Mexican immigrants living in different regions of the US.

## Conclusion

The persistence of ethnomedicinal dietary practices among Mexican immigrants highlights the important role of traditional foods used as medicine across migration contexts. Incorporating these medicinal foods into culturally-centered nutrition programs and interventions, such as FIM, may support chronic disease prevention among Mexican immigrants in the US while promoting transmission of health-related cultural knowledge and connections among Mexican Americans.

## Data Availability

The original contributions presented in the study are included in the article/Supplementary material, further inquiries can be directed to the corresponding author.

## Footnotes

1
www.flourish.studio
