# Supplementary material for: Binational patterns in use of Food is Medicine among Mexican immigrants
Source: Front Nutr. 2026 Apr 24;13:1810991. doi: 10.3389/fnut.2026.1810991 (PMC13152735; doi:10.3389/fnut.2026.1810991)
Supplement: Supplementary file 1 [file Data_Sheet_1.PDF]

Supplemental Table 1. Plant-based Food and Beverages with Scientific Names, Botanical Authorities, and Family Classification

Common Name	Scientific Name	Family
Soups and Stews		
Chicken		
Red Meat		
Cheese		
Vegetable		
Pasta		
Marisco		
Other Soup		
Cold Drinks		
Rice milk	*Oryza sativa* L.	Poaceae
Lemon	*Citrus limon* (L.) Osbeck	Rutaceae
Hibiscus	*Hibiscus sabdariffa* L	Malvaceae
Tamarind	*Tamarindus indica* L.	Fabaceae
Papaya	*Carica papaya* L.	Caricaceae
Other cold drink		
Hot Drinks		
Chamomile	*Matricaria chamomilla* L.	Asteraceae
Mint	*Mentha* L.	Lamiaceae
Spearmint	*Mentha spicata* L.	Lamiaceae
Corn-based drink	*Zea mays* L.	Poaceae
Other hot drink		
Meat		
Beef		
Chicken		
Organ meat		
Other meat		
Seafood		
Cereal		
Corn	*Zea mays* L.	Poaceae
Oats	*Avena sativa* L.	Poaceae
Rice	*Oryza sativa* L.	Poaceae
Wheat	*Triticum aestivum* L.	Poaceae
Other cereals		
Vegetables		
Greens		
Potatoes	*Solanum tuberosum* L.	Solanaceae
Onion	*Allium cepa* L.	Amaryllidaceae
Garlic	*Allium sativum* L.	Amaryllidaceae
Beets	*Beta vulgaris* L.	Amaranthaceae
Squash	*Cucurbita spp.* L.	Cucurbitaceae

Nopales (cactus)	*Opuntia ficus-indica* (L.) Mill.	Cactaceae
Salad		
Other vegetable		
Fruit		
Lemon	*Citrus limon* (L.) Osbeck	Rutaceae
Papaya	*Carica papaya* L.	Caricaceae
Orange	*Citrus sinensis* (L.) Osbeck	Rutaceae
Other fruit		
Legumes		
Beans	*Phaseolus vulgaris* L.	Fabaceae
Lentils	*Lens culinaris* Medik.	Fabaceae
Chickpea	*Cicer arietinum* L.	Fabaceae
Other legume		
Herbs		
Animal-based Foods		
Honey		
Eggs		
Dairy Products (milk, cheese, yogurt)		
Gelatin/Jello		
Other animal-based foods		

Supplemental Table 2. Pairwise comparisons of the foods used among Mexican Immigrants to prevent and treat illness pre- and post-migration by biological sex

Foods		Biological Sex Difference						
English	Spanish	Female			Male			P-value[b]
		n (%)			n (%)			
		Mexico (n=188)	Arizona (n=184)	P-value[a]	Mexico (n=40)	Arizona (n=36)	P-value[a]	
Soups and Stews	***Sopas y Caldos***	143(76.1)	135(73.4)	0.55	30(75.0)	25(69.4)	0.56	0.82
Chicken	Pollo	109(58.0)	96(52.2)	0.19	24(60.0)	18(50.0)	0.16	
Red Meat	Carne	14(7.4)	15(8.2)	0.80	1(2.5)	2(5.6)		
Cheese	Queso	2(1.1)	4(2.2)	0.50	0(0.0)	0(0.0)		
Vegetable	Verdura	19(10.1)	16(8.7)	0.61	3(7.5)	2(5.6)		
Pasta	Fideo	2(1.1)	2(1.1)	>.99	0(0.0)	0(0.0)		
Marisco	Marisco	1(0.5)	2(1.1)	>.99	2(5.0)	0(0.0)		
Other Soups	Otras sopas	34(18.1)	38(20.7)	0.47	7(17.5)	7(19.4)	>.99	
Cold Drinks	***Bebidas Frias***	16(8.5)	15(8.2)	0.74	1(2.5)	3(8.3)	>.99	0.30
Rice milk	Leche de Arroz/Horchata	5(2.7)	4(2.2)	>.99	0(0.0)	0(0.0)		
Lemon	Limon	2(1.1)	1(0.5)	>.99	0(0.0)	0(0.0)		
Hibiscus	Jamaica	1(0.5)	0(0.0)		0(0.0)	0(0.0)		
Tamarind	Tamarindo	0(0.0)	0(0.0)		0(0.0)	0(0.0)		
Papaya	Papaya	1(0.5)	1(0.5)		0(0.0)	0(0.0)		
Other cold drinks	Ortas bebidas frias	7(3.7)	11(6.0)	0.38	1(2.5)	3(8.3)		
Hot Drinks	***Bebidas Calientes***	9(4.8)	10(5.4)	0.76	2(5.0)	1(2.8)		0.60
Chamomile	Manzanilla	1(0.5)	0(0.0)		0(0.0)	0(0.0)		
Mint	Menta	1(0.5)	0(0.0)		0(0.0)	0(0.0)		
Spearmint	Hierbabuena	0(0.0)	2(1.1)		0(0.0)	0(0.0)		
Corn-based drink	Atole/champurrado/pinole	5(2.7)	2(1.1)	0.50	1(2.5)	1(2.8)		
Other hot drinks	Ortas bebidas calientes	4(2.1)	7(3.8)	0.73	1(2.5)	0(0.0)		
Meat	***Carne***	27(14.4)	26(14.1)	0.82	5(12.5)	5(13.9)	>.99	0.87
Beef	Carne de res	9(4.8)	6(3.3)	0.51	2(5.0)	1(2.8)	>.99	
Chicken	Pollo	13(6.9)	16(8.7)	0.58	2(5.0)	4(11.1)	>.99	
Organ meat	Vísceras	3(1.6)	1(0.5)	0.50	1(2.5)	1(2.8)		
Other meats	Otras carnes	5(2.7)	7(3.8)	0.73	1(2.5)	0(0.0)		
Seafood	***Marisco***	13(6.9)	15(8.2)	>.99	4(10.0)	3(8.3)	0.50	0.61
Cereal	***Cereales***	20(10.6)	19(10.3)	>.99	3(7.5)	2(5.6)		0.80
Corn	Maiz	2(1.1)	2(1.1)	>.99	0(0.0)	0(0.0)		
Oats	Avena	8(4.3)	5(2.7)	0.50	1(2.5)	1(2.8)		
Rice	Arroz	10(5.3)	8(4.3)	0.69	1(2.5)	1(2.8)		
Wheat	Trigo	1(0.5)	1(0.5)	>.99	0(0.0)	0(0.0)		
Other cereals	Otros cereales	0(0.0)	3(1.6)	>.99	1(2.5)	0(0.0)		
Vegetables	***Verduras***	77(41.0)	69(37.5)	0.40	10(25.0)	11(30.6)	>.99	0.46
Greens	Quelite	11(5.9)	6(3.3)	0.29	3(7.5)	1(2.8)		
Potatoes	Papas	8(4.3)	5(2.7)	0.45	1(2.5)	1(2.8)		
Onion	Cebolla	7(3.7)	6(3.3)	>.99	0(0.0)	0(0.0)		
Garlic	Ajo	4(2.1)	6(3.3)	0.63	0(0.0)	0(0.0)		
Beets	Betabel	4(2.1)	2(1.1)	0.50	0(0.0)	0(0.0)		
Squash	Calabaza/Chayote	1(0.5)	3(1.6)	0.50	0(0.0)	0(0.0)		
Nopales (cactus)	Nopales	10(5.3)	7(3.8)	>.99	3(7.5)	2(5.6)	>.99	
Salad	Ensalada	7(3.7)	10(5.4)	0.79	1(2.5)	2(5.6)		
Other vegetables	Otras verduras	51(27.1)	50(27.2)	>.99	5(12.5)	8(22.2)	0.50	
Fruit	***Fruta***	27(14.4)	28(15.2)	0.45	7(17.5)	5(13.9)	0.25	0.52
Lemon	Limon	7(3.7)	3(1.6)	>.99	0(0.0)	0(0.0)		

Papaya	Papaya	2(1.1)	2(1.1)	0.38	1(2.5)	0(0.0)		
Orange	Naranja	6(3.2)	2(1.1)	0.06	0(0.0)	0(0.0)		
Other fruits	Otras frutas	17(9.0)	24(13.0)	0.65	7(17.5)	5(13.9)	0.25	
Legumes	***Legumbre***	33(17.6)	31(16.8)	0.15	6(15.0)	3(8.3)	0.50	0.43
Beans	Frijoles	24(12.8)	19(10.3)	0.15	4(10.0)	2(5.6)	>.99	
Lentils	Lentejas	11(5.9)	17(9.2)	0.25	2(5.0)	1(2.8)		
Chickpea	Garbanzo	2(1.1)	5(2.7)	0.25	0(0.0)	0(0.0)		
Other legumes	Otros legumbres	0(0.0)	0(0.0)		0(0.0)	0(0.0)		
Herbs	***Hierbas***	15(8.0)	7(3.8)	**0.04**	0(0.0)	0(0.0)		
Animal-based Foods	***Comidas de Animales***	20(10.6)	21(11.4)	>.99	4(10.0)	4(11.1)	>.99	0.92
Honey	Miel	9(4.8)	8(4.3)	>.99	0(0.0)	1(2.8)		
Eggs	Huevos	5(2.7)	3(1.6)	>.99	1(2.5)	1(2.8)		
Dairy Products (milk, cheese, yogurt)	Productos Lactivos (leche, queso, yogur)	4(2.1)	4(2.2)		1(2.5)	0(0.0)		
Gelatin/Jello	Gelatina	3(1.6)	7(3.8)	0.38	3(7.5)	2(5.6)		
Other animal-based foods	Otras Comidas de Animales	0(0.0)	0(0.0)		0(0.0)	0(0.0)		

[a] P-values were obtained from McNemar test or McNemar exact test for low counts for pairwise comparisons

[b] P-values were obtained from generalized linear mixed models with repeated measures

Supplemental Table 3. Pairwise comparisons of the foods used among Mexican Immigrants to prevent and treat illness pre- and post-migration by age prior to migrating to the US

Foods		Age at time of migration						
English	Spanish	<30 years			≥ 30 years			P-value[b]
		n (%)			n (%)			
		Mexico (n=129)	Arizona (n=132)	P-value[a]	Mexico (n=99)	Arizona (n=88)	P-value[a]	
Soups and Stews	***Sopas y Caldos***	99(76.7)	96(72.7)	0.47	74(74.7)	64(72.7)	0.76	0.80
Chicken	Pollo	76(58.9)	68(51.5)	0.21	57(57.6)	46(52.3)	0.61	
Red Meat	Carne	8(6.2)	9(6.8)	>.99	7(7.1)	8(9.1)	>.99	
Cheese	Queso	2(1.6)	3(2.3)	>.99	0(0.0)	1(1.1)	>.99	
Vegetable	Verdura	13(10.1)	13(9.8)	>.99	9(9.1)	5(5.7)	0.73	
Pasta	Fideo	0(0.0)	1(0.8)	>.99	2(2.0)	1(1.1)	>.99	
Marisco	Marisco	1(0.8)	1(0.8)	>.99	2(2.0)	1(1.1)		
Other Soups	Otras sopas	24(18.6)	27(20.5)	0.68	17(17.2)	18(20.5)	0.76	
Cold Drinks	***Bebidas Frias***	10(7.8)	13(9.8)	0.69	7(7.1)	5(5.7)	0.63	0.46
Rice milk	Leche de Arroz/Horchata	3(2.3)	3(2.3)	>.99	2(2.0)	1(1.1)	>.99	
Lemon	Limon	0(0.0)	0(0.0)		1(1.0)	0(0.0)		
Hibiscus	Jamaica	2(1.6)	1(0.8)		0(0.0)	0(0.0)		
Tamarind	Tamarindo	0(0.0)	0(0.0)		1(1.0)	0(0.0)		
Papaya	Papaya	0(0.0)	0(0.0)		0(0.0)	0(0.0)		
Other cold drinks	Ortas bebidas frias	1(0.8)	1(0.8)		0(0.0)	0(0.0)		
Hot Drinks	***Bebidas Calientes***	4(3.1)	10(7.6)	0.13	4(4.0)	4(4.5)	>.99	
Chamomile	Manzanilla	6(4.7)	6(4.5)	>.99	5(5.1)	5(5.7)	>.99	0.87
Mint	Menta	0(0.0)	0(0.0)		1(1.0)	0(0.0)		
Spearmint	Hierbabuena	0(0.0)	0(0.0)		1(1.0)	0(0.0)		
Corn-based drink	Atole/champurrado/pinole	0(0.0)	1(0.8)		0(0.0)	1(1.1)		
Other hot drinks	Ortas bebidas calientes	4(3.1)	2(1.5)	0.39	0.50	1(1.1)	0.39	
Meat	***Carne***	2(1.6)	4(3.0)	0.43	>.99	3(3.4)	>.99	
Beef	Carne de res	16(12.4)	17(12.9)	0.91	>.99	14(15.9)	>.99	0.93
Chicken	Pollo	4(3.1)	4(3.0)	0.97	>.99	3(3.4)	0.22	
Organ meat	Vísceras	9(7.0)	14(10.6)	0.30	0.55	6(6.8)	>.99	
Other meats	Otras carnes	2(1.6)	2(1.5)		2(2.0)	0(0.0)		
Seafood	***Marisco***	3(2.3)	0(0.0)		3(3.0)	7(8.0)	0.06	
Cereal	***Cereales***	9(7.0)	8(6.1)	0.45	8(8.1)	10(11.4)	0.73	0.44
Corn	Maiz	12(9.3)	10(7.6)	0.73	11(11.1)	11(12.5)	0.69	0.55
Oats	Avena	1(0.8)	1(0.8)	>.99	1(1.0)	1(1.1)		
Rice	Arroz	3(2.3)	2(1.5)	>.99	6(6.1)	4(4.5)	>.99	
Wheat	Trigo	8(6.2)	7(5.3)	>.99	3(3.0)	2(2.3)		
Other cereals	Otros cereales	0(0.0)	0(0.0)		1(1.0)	1(1.1)		
Vegetables	***Verduras***	0(0.0)	0(0.0)		1(1.0)	3(3.4)		
Greens	Quelite	53(41.1)	45(34.1)	0.06	34(34.3)	35(39.8)	0.33	0.16
Potatoes	Papas	10(7.8)	3(2.3)	**0.02**	4(4.0)	4(4.5)	0.50	
Onion	Cebolla	6(4.7)	3(2.3)	0.45	3(3.0)	3(3.4)		
Garlic	Ajo	6(4.7)	6(4.5)	>.99	1(1.0)	0(0.0)		
Beets	Betabel	3(2.3)	5(3.8)	0.50	1(1.0)	1(1.1)	>.99	
Squash	Calabaza/Chayote	1(0.8)	0(0.0)	0.50	3(3.0)	2(2.3)	>.99	
Nopales (cactus)	Nopales	1(0.8)	2(1.5)	>.99	0(0.0)	1(1.1)		
Salad	Ensalada	11(8.5)	6(4.5)	0.29	2(2.0)	3(3.4)	0.50	
Other vegetables	Otras verduras	2(1.6)	8(6.1)	0.13	6(6.1)	4(4.5)	0.73	
Fruit	***Fruta***	32(24.8)	30(22.7)	0.42	24(24.2)	28(31.8)	0.18	
Lemon	Limon	17(13.2)	18(13.6)	>.99	17(17.2)	15(17.0)	>.99	0.88

Papaya	Papaya	5(3.9)	3(2.3)	0.69	2(2.0)	0(0.0)		
Orange	Naranja	3(2.3)	1(0.8)	>.99	0(0.0)	1(1.1)		
Other fruits	Otras frutas	2(1.6)	0(0.0)	>.99	4(4.0)	2(2.3)	0.63	
Legumes	***Legumbre***	12(9.3)	15(11.4)	0.58	12(12.1)	14(15.9)	0.51	
Beans	Frijoles	19(14.7)	21(15.9)	>.99	20(20.2)	13(14.8)	0.11	0.31
Lentils	Lentejas	14(10.9)	14(10.6)	>.99	14(14.1)	7(8.0)	0.02	
Chickpea	Garbanzo	6(4.7)	11(8.3)	0.29	7(7.1)	7(8.0)	>.99	
Other legumes	Otros legumbres	0(0.0)	3(2.3)		2(2.0)	2(2.3)		
Herbs	***Hierbas***	0(0.0)	0(0.0)		0(0.0)	0(0.0)		
Animal-based Foods	***Comidas de Animales***	11(8.5)	7(5.3)	0.29	4(4.0)	0(0.0)		
Honey	Miel	11(8.5)	13(9.8)	>.99	13(13.1)	12(13.6)	>.99	0.86
Eggs	Huevos	6(4.7)	6(4.5)	>.99	3(3.0)	3(3.4)	>.99	
Dairy Products (milk, cheese, yogurt)	Productos Lactivos (leche, queso, yogur)	1(0.8)	1(0.8)		5(5.1)	3(3.4)	>.99	
Gelatin/Jello	Gelatina	2(1.6)	2(1.5)		3(3.0)	2(2.3)		
Other animal-based foods	Otras Comidas de Animales	3(2.3)	5(3.8)	>.99	3(3.0)	4(4.5)	0.50	
Soups and Stews	***Sopas y Caldos***	0(0.0)	0(0.0)		0(0.0)	0(0.0)		

[a] P-values were obtained from McNemar test or McNemar exact test for low counts for pairwise comparisons

[b] P-values were obtained from generalized linear mixed models with repeated measures
